# Genomic Selection for Pea Grain Yield and Protein Content in Italian Environments for Target and Non-Target Genetic Bases

**DOI:** 10.3390/ijms26072991

**Published:** 2025-03-25

**Authors:** Margherita Crosta, Nelson Nazzicari, Luciano Pecetti, Tommaso Notario, Massimo Romani, Barbara Ferrari, Giovanni Cabassi, Paolo Annicchiarico

**Affiliations:** Research Centre for Animal Production and Aquaculture, Council for Agricultural Research and Economics, 26900 Lodi, Italy; nelson.nazzicari@crea.gov.it (N.N.); luciano.pecetti@crea.gov.it (L.P.); tommaso.notario@crea.gov.it (T.N.); barbara.ferrari@crea.gov.it (B.F.); giovanni.cabassi@crea.gov.it (G.C.)

**Keywords:** crop quality, crude protein yield, genetic base, genome-enabled prediction, GWAS, inter-population prediction, *Pisum sativum*

## Abstract

Enhanced pea cultivation, which can increase the sustainability of European agriculture, requires better-performing cultivars. This study investigated the genomic selection (GS) ability to predict grain yield, protein content, and protein yield on the same or a different genetic base (target/non-target GB) relative to that employed for model training. GS models were developed on 276 lines from three Recombinant Inbred Line (RIL) populations evaluated in three Italian autumn-sown environments using 5537 SNPs from genotyping by sequencing. Validation in two cropping years concerned 108 independent lines from five RIL populations, of which two belonged to the GS training set, and three shared one parent each with training populations. A genome-wide association study performed on the GS training set using 18,674 SNPs highlighted the polygenic control of protein content and grain yield, with several environment-dependent QTLs for yield. Intermediate/high predictive ability within or across populations emerged for all traits in the target GB (0.359–0.675), with some variation depending on the population. Predictive ability in the non-target GB was modest/intermediate for protein content, and null/poor for the other traits. No inverse correlation emerged between grain yield and protein content. GS proved useful for all traits in the target GB and for protein content in a non-target GB.

## 1. Introduction

Enhancing legume cultivation is fundamental to improving the sustainability of European agriculture and to reducing its massive deficit of high-protein feedstuff [1]. Legume cropping can have a positive impact on several aspects, including energy and resource use efficiency, greenhouse gas emissions, nitrogen biogeochemical fluxes, and agricultural biodiversity [2,3,4]. The European Union (EU) has supported grain legume cultivation by various measures contained in the Common Agricultural Policy, but the diffusion of these crops is still severely hampered by their lower profitability relative to the main cereal crops [5,6].

Field pea (*Pisum sativum* L.) revealed a trend toward higher yield than other autumn-sown grain legumes in western [7] and southern Europe [8], along with a good rate of genetic yield progress [9]. However, its low yield compared with major cereals [2,10] and its yield instability due to biotic and abiotic stresses [11,12] have established grain yield enhancement as the primary breeding goal. Grain yield improvement is challenged by a remarkable genotype × environment interaction (GEI), which was often reported especially in southern Europe [13,14]. Moreover, the relatively low grain protein content of pea relative to other grain legumes (usually in the range of 22–26% on a dry-matter basis vs. around 40% for both soybean and white lupin [15]) makes protein enhancement a major breeding objective for both animal feeding and human consumption [16,17]. Improvement in this trait is facilitated by lower GEI relative to grain yield [18] and the absence of inverse genetic correlation with grain yield according to various reports [18,19,20,21]. Anyway, grain yield affected protein yield per unit area (as determined by the product of grain yield and protein content) much more than protein content in earlier studies in Italy [18,22].

Several studies reported quantitative trait loci (QTLs) for pea grain yield or its components [18,23,24,25,26,27,28,29,30,31] and protein content [18,23,24,25,26,27,29,30,31,32]. The large number of detected QTLs and the generally small proportion of variance explained by each of them suggested the polygenic control of these traits. Genomic selection (GS), which uses a statistical model based on many genome-wide molecular markers to predict breeding values [33], can be more convenient than marker-assisted selection for trait improvement in this context [34]. The adoption of GS has the potential to reduce the length of the breeding cycle by at least 50% relative to phenotypic selection and decrease remarkably the cost per cycle by diminishing the need for field-based selection [35]. The recent sequencing of the pea genome [36] and the development of high-throughput genotyping techniques, such as genotyping by sequencing (GBS; [37]), have facilitated the application of GS to pea. Previous studies encouraged the genomic prediction of pea grain yield or its components in different environments and plant materials based on predictive ability values alone [38,39,40] or higher predicted efficiency relative to phenotypic selection [20,41,42,43]. Pioneering studies were also encouraging for protein content genomic prediction, showing a predictive ability higher than [18,31] or comparable [43] to that of grain yield. In addition, GS displayed superior predicted efficiency relative to phenotypic selection for improvement in protein yield [18]. Other target traits for successful GS application in pea were the resistance to biotic stresses [44,45] and the optimization of seed mineral concentration [46].

Despite its potential interest, GS requires time and resources for the definition of prediction models for a given genetic base (target GB), e.g., a specific breeding population. The ability of a model defined for a target GB to predict breeding values in another GB (non-target GB) is of considerable practical interest for breeding programs to optimize the phenotyping work required for model development. Indeed, a quantification of the predictive ability loss in an inter-population scenario relative to an intra-population scenario has been investigated in major inbred crops, such as soybean [47,48] and wheat [49]. Preliminary assessments for pea indicated a substantial loss of predictive ability that was more pronounced for grain yield than for protein content [18,20,31,41,42].

The objectives of this study were to (a) investigate the genetic control of grain yield and protein content by a GWAS, considering the effect of different environmental conditions on grain yield QTLs; and (b) quantify the GS predictive ability in a target and a non-target GB for grain yield, protein content, and protein yield based on a higher number of populations and environments compared with previous studies in pea. In this context, this study validated GS models for the three traits of interest trained on the same materials employed for the GWAS, i.e., three Recombinant Inbred Line (RIL) populations created by connected crosses between three elite cultivars (target GB), evaluated in three Italian autumn-sown environments. The validation, based on two Italian autumn-sown environments, was performed on (a) independent lines from two RIL populations belonging to the target GB, and (b) lines from a non-target GB represented by three other RIL populations, each sharing one parent with the populations of the target GB.

## 2. Results

### 2.1. Phenotypic Data Analysis of the Genomic Selection Validation Set

For all traits in the GS validation set, the variation due to the RIL population, cropping year, line within population, and line within population × year interaction was significant (*p* < 0.05), while population × year interaction was not significant (*p* > 0.10) (Table S1). Broad-sense heritability values for each GB in each validation cropping year, which were high for protein content (0.74–0.91) and intermediate or high for grain yield (0.54–0.77) and protein yield (0.62–0.79), are reported in Table S2. The non-target GB tended to display somewhat higher broad-sense heritability values than the target GB in both cropping years as shown in Table S2, in accordance with its higher number of populations (three vs. two) and parent lines (five vs. three). All traits showed higher mean values in 2018–2019 relative to 2019–2020, especially grain yield (+33%) and protein yield (+44%) but also, to a lower extent, seed protein content (+8%). Table 1 reports for each trait and RIL population the mean value across cropping years, the phenotypic coefficient of variation (*CV*) in each year, and the phenotypic correlation (*r*) of line values across the two cropping years (a high value of which indicates low line × year interaction). Attika × Isard (A × I) and Kaspa × Isard (K × I) showed superior grain and protein yield relative to the other RIL populations, while Attika × Guifilo (A × G) displayed the highest protein content. Alliance × Isard (C × I) was in the bottom group of means for all traits. In general, *CV* values were much higher for grain and protein yield relative to protein content. Wide differences in *CV* values between cropping years were observed especially for protein content in C × I. The phenotypic correlation of line values across years was mostly significant (*p* < 0.05) but relatively low, especially for C × I (which was, therefore, particularly subject to GEI). On average, grain yield was more affected by GEI than protein content or protein yield according to mean correlation values (0.38 vs. 0.52 and 0.46; Table 1).

A slightly positive, non-significant correlation between grain yield and protein content (*r* = 0.14, *p* > 0.10) was observed for trait values averaged across the two cropping years.

### 2.2. Genome-Wide Association Study

**Figure 1:**
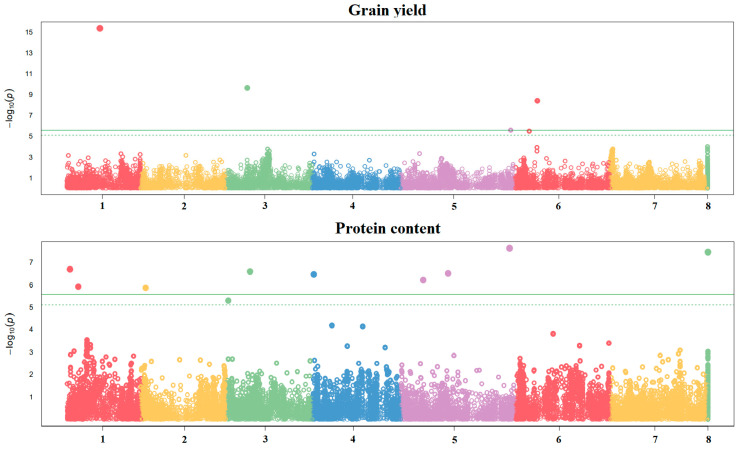
Manhattan plots showing the association scores of 18,674 SNPs along the 7 pea chromosomes (chromosome 8 represents scaffolds) with two traits averaged across three test environments. The GWAS was based on the Blink model and 276 lines belonging to three connected Recombinant Inbred Line populations. The continuous and dashed lines represent Bonferroni and False Discovery Rate thresholds at 1%, respectively.

The GWAS performed on the GS training set based on 18,674 Single-Nucleotide Polymorphisms (SNPs) revealed several significant associations for grain yield and protein content mean values, confirming the definite polygenic control of these traits. Five significant SNPs mapped on chromosomes 1, 3, 5, and 6 were found for grain yield, whereas ten significant SNPs were identified for protein content, of which nine mapped on chromosomes 1, 2, 3, 4, and 5 and one on scaffolds (Figure 1).

The significant SNPs for grain yield were largely environment-dependent, which was likely due to differences in winter cold stress intensity between environments. This finding emerged when comparing the results obtained for the same location (Lodi) in a mild-winter cropping year (2013–2014, featuring an absolute minimum temperature of −5.7 °C) vs. a cold-winter year (2014–2015, with an absolute minimum temperature of −11.6 °C). Four significant SNPs were detected on chromosomes 2, 3, and 6 in Lodi in 2013–2014, and eight were detected on chromosomes 1, 2, 3, 5, and 6 in Lodi in 2014–2015 (Figure S1). The cold environment had a predominant influence on line mean grain yield, as confirmed by the fact that all the significant SNPs detected for mean grain yield, except for one on chromosome 6, were either significant or close to significant SNPs in this environment (Figure 1 and Figure S1).

The list of significant SNPs detected for grain yield and protein content is provided in Table S3 along with their estimated effect, while a list of the candidate genes relative to the analysis performed on trait mean values is provided in Table S4.

### 2.3. Genomic Selection

After filtering, the GS validation set retained 3460 polymorphic SNPs for A × I, 4487 for K × I, 2981 for D × A, 3217 for A × G, and 3848 for C × I. Predictive ability values for the target and non-target GB are presented in Table 2 for both within- and across-population predictions. The GS models validated on the target GB displayed moderately high predictive ability both within and across populations for all traits (0.359–0.675). Higher predictions emerged for the validation performed on 2019–2020 data (0.458–0.675) relative to 2018–2019 (0.359–0.560) in the target GB, which, for grain and protein yield, could be explained by the higher heritability characterizing 2019–2020 (Table S2). For the non-target GB, predictions were very poor or null in all cases except for protein content, which showed intermediate within-population predictive ability (0.314–0.372) and modest across-population predictions (0.117–0.295). The predictive ability values within RIL populations tended to be higher (from −0.089 to 0.372) than those across populations (from −0.269 to 0.295) for the non-target GB. Protein content showed the highest predictive ability in all the scenarios (as defined by the combination of the GB, validation data, and prediction type), in agreement with the generally superior heritability of this trait (Table S2). As a result, protein yield predictions were higher than grain yield ones in the target GB, independently of the predictive ability estimation method (Table 2).

Predictive ability results for each RIL population in the target and non-target GB are reported in Table 3. For grain and protein yield in the target GB, higher predictive ability was found for K × I relative to A × I, independently from the validation dataset. In the non-target GB, C × I showed the highest predictive ability for grain yield, and, usually, the lowest predictions for protein content. In some cases, the predictive ability was largely affected by the validation year, as for protein content in the populations A × G and K × I, and protein yield in C × I (Table 3).

## 3. Discussion

The higher mean values of grain and protein yield observed in 2018–2019 relative to 2019–2020 could be attributed to the much higher rainfall amount in the former cropping year (Table S5). For grain yield, substantial GEI within RIL populations emerged from linear mixed model analysis results and the correlation of line values across cropping years, in agreement with previous studies for southern Europe [13,14,18]. Seed protein content was affected by GEI to a lower extent. The much higher variation within RIL populations observed for grain yield relative to protein content confirms earlier results for breeding material [18]. For grain and protein yield, the inconsistency between GS validation cropping years for extent of within-population variation (as estimated by CV values) that was observed in some cases (i.e., K × I for both traits, and C × I for grain yield) might be due to different population responses to drought (mainly occurring in the second cropping year) and winter cold stress (mainly occurring in the first cropping year). The absence of inverse correlation between grain yield and protein content, which facilitates the simultaneous improvement of both traits, agrees with several previous reports [18,19,21,31] but not all of them [24,26].

GWAS results highlighted the definite polygenic control of grain yield and protein content by revealing many significant markers spread across the genome for trait mean data. These findings confirms the importance of developing GS models for both traits and their combination.

Within-population and across-population predictions were assessed for the target and non-target GB. The former are meaningful when the main goal of GS is the identification of the best genotypes within a specific RIL population, whereas the latter are relevant when GS aims to detect the best lines across several RIL populations. Overall, within-RIL population predictive ability values for the target GB were moderately high, on average slightly higher than those reported earlier for the same traits and similar material [18]. For protein content, within-population and across-population predictions for the non-target GB suffered a substantial penalty relative to the target GB, while grain and protein yield predictions dropped to zero in the non-target GB. Possible reasons contributing to the predictive ability drop when moving from the target to the non-target GB are differences in QTL effect between the training and validation sets, and the absence of allelic variation in the training set for QTLs that are relevant for the validation set. These factors may also impair the GS model ability to predict population trait mean values in the non-target GB, as suggested by within-population predictions exceeding across-population ones in this material set. These findings agree with those of a previous pea study, in which GS models trained on a world germplasm collection (mainly consisting of landrace material) and validated on three RIL populations displayed moderate across-population predictive ability for protein content and null predictions for grain yield [31]. A preliminary study of GS inter-population prediction, using just one RIL population as a training set, revealed a less dramatic drop in predictive ability for grain and protein yield than the present study [18]. A slight difference between intra- and inter-population predictive ability was reported for grain yield and protein content of soybean [47,48]. However, in the former study, the validation GB was included in the training set also in the inter-population scenario.

In general, within-population predictive ability values were moderately consistent across years for each population. Some inconsistencies could be related to the different extent of within-population variation between years (as estimated by CV). For instance, the predictive ability for the protein content of the A × G population in 2018–2019 was over two-fold that in 2019–2020 (Table 1), in agreement with the about two-fold CV value in the former year relative to the latter (Table 3). The high GEI of lines within populations could have been another factor contributing to predictive ability differences between cropping years. For example, the contrasting predictive ability observed between years for protein yield of the population C × I (Table 3) was associated with the non-significant correlation of line values across years (Table 1). A factor possibly contributing to differences in predictive ability between populations was the number of polymorphic SNPs, especially in a context, such as our GS, characterized by a modest marker number relative to the LD decay pattern. Indeed, the better predictive ability for grain and protein yield displayed by population K × I relative to A × I was associated with a greater number of polymorphic SNPs in the former population (4487 vs. 3460). Moreover, K × I likely benefitted from a higher number of lines included in the GS training set relative to A × I (103 vs. 77). The within-population predictive ability values observed for grain yield in the non-target GB, despite being quite low, reflected the number of polymorphic SNPs in each population. Such a trend did not emerge for protein content, possibly suggesting a lower number of QTLs involved in its determination relative to grain yield, implying a lower prediction benefit from marker number increase. The modest or intermediate within-population predictive ability found for protein content in the non-target GB, which contrasts with the null or poor predictions observed for grain yield, was already reported for a very different GB [31]. This result suggests that the QTLs for protein content may be less dependent on the specific population considered, in terms of position and effect, than the QTLs for grain yield. This could possibly facilitate protein content prediction in a non-target GB relative to grain yield.

Earlier work comparing GS and phenotypic selection in terms of predicted protein yield gains per unit time for a similar budget suggested the convenience of GS when its predictive ability exceeds 0.25 [18]. This threshold was always exceeded for the target GB in this study.

Differently from GS, which relied on 5537 common SNPs between the training and validation sets, the GWAS was based on all 18,674 SNPs available for the GS training set, which ensured a good genome coverage relative to the LD decay pattern. Its results highlighted the different genetic control of grain yield depending on the intensity of winter cold stress. With respect to our GWAS results for trait mean values, previous studies detected QTLs in the same genomic regions as our significant SNPs on chromosomes 1 [29], 5 [27,28], and 6 for grain yield [30,31], and on chromosomes 2 [28], 3 [29], 4 [27], and 5 for protein content [27,28,29,30]. Many candidate genes of possible interest emerged for both grain yield and protein content due to the slow LD decay, which impeded the identification of single candidate genes for the significant SNPs. For grain yield, Psat1g096760 encodes a phosphatidylethanolamine-binding protein that can be involved in flowering control in response to environmental conditions [50], while Psat3g051840, Psat3g051880, and Psat5g289760 code for transcription factors whose families (RING for the first two, and BZIP for the last one) play a role both in plant growth and abiotic stress response [51,52]. Moreover, Psat5g289640 encodes an electron transfer flavoprotein that regulates the flux to the mitochondrial transport chain under carbohydrate-limiting conditions [53]. For protein content, Psat5g132320 may participate in plant symbiosis with *Rhizobium leguminosarum*, since it encodes a lysin motif domain that is known to play a key role in plant–microbe interaction [54], whereas Psat2g022320 codes for an ethylene-insensitive 3 protein that is involved in leaf senescence and nitrogen metabolism in wheat [55].

## 4. Materials and Methods

### 4.1. Plant Material and Phenotyping

The GS training set included 276 genotypes belonging to three RIL populations created by connected crosses between three elite cultivars, i.e., the European cultivars Attika and Isard, and the Australian cultivar Kaspa (indicated by the initials A, I, and K, respectively). These populations were indicated by A × I, K × A, and K × I, and consisted of 77, 96, and 103 lines, respectively. These materials were evaluated for grain yield and protein content in three environments with autumn-sown crops of northern or central Italy, of which two were managed organically (Lodi 2013–2014 and Perugia 2013–2014) and one conventionally (Lodi 2014–2015), by using a randomized complete block design with three replicates [18,41]. Lodi is located in northern Italy and features a subcontinental climate and sandy-loam soil, while Perugia is in central Italy and is characterized by a cool Mediterranean climate and a silty-clay-loam soil. Grain yield was determined, after combine harvesting, by assessing seed moisture on a random sample of 250 seeds oven-dried at 90 °C for four days. The GS validation set consisted of genotypes not included in the GS training set from five RIL populations, of which two represented the target GB (A × I and K × I, consisting of 19 and 22 lines, respectively) and three the non-target GB. The RIL population K × A, which was used for GS model training, was excluded from the validation set because of its lower agronomic value in terms of grain yield and frost tolerance [41]. Each RIL population of the non-target GB had one parent in common with the target GB and one that was different. The different parents were represented by the French cultivars Alliance and Dove, and the Spanish cultivar Guifilo (indicated by the letters C, D, and G, respectively), generating the RIL populations D × A, A × G, and C × I. The first two populations consisted of 23 lines each, while the third one consisted of 21 lines. The parent lines were selected from a larger group of international cultivars because of their high and stable grain yield and the moderate phenological differences between the environments of northern and southern Italy [13,56]. The widespread use of Attika as a parent in the non-target GB was due to its good competitive ability against weeds [57], which is crucial especially under organic management. The validation set was evaluated in Lodi during autumn sowing in the cropping years 2018–2019 and 2019–2020. Each of these two experiments was organized as a split plot featuring three replicates, with the growing condition (mixed or pure stand) on the main plot and the pea genotype on the subplot, but only pure stand data, analyzed in a randomized complete block design, were employed for GS validation in this study [58]. Dry grain yield was determined, after combine harvesting, by assessing seed moisture on a random sample of 100 seeds oven-dried at 90 °C for 4 days. The grain protein content of both the GS training and validation sets was determined by near-infrared spectroscopy (NIRS) on 100 g of dry seed per plot, ground by a cutting mill with a 1 mm mesh sieve, by a Nirflex 500 spectrometer (Büchi, Cornaredo, Italy) working in the 1000–2500 nm range. The reference data were obtained by the analysis of total nitrogen content by Dumas’s method with a ThermoQuest NA1500 elemental analyzer (Carlo Erba, Milano, Italy) and atropine as a standard. The Partial Least Squares method within PLS Toolbox 8.9 (Eigenvector Research Inc., Washington, DC, USA) was employed to develop a prediction model, featuring an *R*^2^ of 0.78, while the calibration *R*^2^ amounted to 0.93. Grain protein content was obtained by multiplying the NIRS-estimated nitrogen content by 6.25. Protein yield was obtained by multiplying grain yield by protein content plot values.

### 4.2. Phenotypic Data Analysis of the Genomic Selection Validation Set

The analyses concerned grain yield, protein content, and protein yield. A linear mixed model with RIL population (*R_k_*), cropping year (*Y_j_*), and their interaction (*R_k_Y_i_*) as fixed factors, and replicate (*B_r_*), line within RIL population (*G_i_(R_k_)*), and line × year interaction (*G_i_(R_k_)Y_j_*) as random factors, was employed to evaluate the significance of these sources of variation, according to the formula

*Y_kjir_* = *m* + *R_k_* + *Y_j_* + *R_k_Y_i_* + *B_r_* + *G_i_(R_k_)* + *G_i_(R_k_)Y_j_* + *e_kjir_*

where *m* is the trait mean and *e_kjir_* the model residual. The presence of significant differences between RIL population means was assessed by Duncan’s test. The broad-sense heritability was estimated for each GB during each cropping year using the variance components relative to genotype ($S_{G}^{2}$) and experimental error ($S_{e}^{2}$), according to the formula

$$H^{2}\;=\;S_{G}^{2}/(S_{G}^{2}+S_{e}^{2}/n)$$

where *n* represents the number of replicates in each experiment. The phenotypic correlation of line values across the two cropping years was estimated for each trait and RIL population, to assess the consistency of line responses across years. All the analyses were performed by R Studio version 4.3.1.

### 4.3. Genotyping and Genomic Data Processing

Detailed information about DNA isolation, GBS, SNP calling, and missing data imputation can be found in [20] for the GS training set, and in [58] for the GS validation set. In summary, GBS was performed according to the protocol in [37] with modifications, sequence alignment was executed on reference genome version 1a [36], SNP calling was conducted by the dDocent pipeline [59], and quality filtering by vcftool [60]. Genomic data of the training set were filtered by minor allele frequency (MAF) > 5%, missing per marker < 20%, missing per sample < 25%, and SNP heterozygosity < 30%. Genomic data of the validation set were filtered by MAF > 5%, missing per marker < 10%, missing per sample < 25%, and SNP heterozygosity < 30%. Missing data imputation was performed by the Random Forest method using the R package MissForest [61] for the GS training set, and by the k-nearest neighbors imputation method [62] for the GS validation set.

### 4.4. Genome-Wide Association Study

A GWAS was performed on the same material employed for GS model training relying on all 18,674 SNPs retained by filtering using the Blink model [63] within the GAPIT R package [64]. Significant SNPs were selected according to a False Discovery Rate threshold at 1%. A GWAS had already been performed on the same dataset [18] with methods that are now obsolete, which is why we decided to repeat it by using updated models (thereby generating quite different results). The GWAS was performed on the mean grain yield and protein content data across the three evaluation environments, and on grain yield data from each of the two cropping years in Lodi, to investigate the effect of different environmental conditions on the QTLs detected in the same location. Indeed, Lodi in 2014–2015 featured much more severe winter cold stress than Lodi in 2013–2014 [41]. The analysis for the single cropping years in Lodi was performed just for grain yield because, differently from protein content, it was largely affected by GEI in previous work [18]. In this study, the GWAS was preferred to composite interval mapping (CIM), which is normally employed to identify marker–trait associations in experimental populations, because it allowed for a joint analysis of all the RIL populations. This ensured a much higher statistical power compared to that achievable by CIM, which would have relied on three separate within-population analyses, each based on about one-third of the total number of individuals [65]. Population structure information to be included in the GWAS model was obtained by a DAPC [66] performed on genotype data pruned for an excess of LD to avoid the strong influence of SNP clusters when estimating genetic relatedness [67]. Pruning was performed on SNPs of known genomic position by the snp.pruning () function from the R package ASRgenomics with a maximum *R*^2^ threshold of 0.2, a window size of 50 SNPs, and an overlap of 5 SNPs between consecutive windows, generating a set of 5094 SNPs. For the DAPC, the k-means clustering algorithm was run iteratively for increasing values of K (i.e., numbers of genetic clusters) from 1 to 30, to identify its optimal value according to differences between successive values of the Bayesian information criterion. The analysis was performed on the output of a principal component analysis (PCA) to benefit from dimensionality reduction while keeping all the PCs to avoid information loss. The final DAPC was performed by using the optimal K value, which was equal to three, in accordance with the number of RIL populations forming this material set. The number of PCs to be retained for DAPC and the number of discriminant functions to be used as covariates in GWAS models were determined by visual inspection of plots of PC cumulative variance and discriminant function eigenvalues, respectively. Based on this operation, 150 PCs were considered for DAPC, and 2 discriminant functions were employed as GWAS covariates, showing appropriate compensation for population structure (Figure S2). The procedure was implemented by using the functions find.clusters () and dapc () from the R package adegenet [68]. LD was estimated as an *R*^2^ value for pairwise combinations of SNPs within a 100 kb window by the LD.decay () function from the R package sommer [69]. The *R*^2^ values were plotted against physical distance and fitted by a polynomial curve as described in [70]. The 90th percentile of the *R*^2^ distribution for pairwise combinations of SNPs located on different chromosomes was estimated by setting the argument unlinked to true in the LD.decay () function, to assess the most meaningful LD decay threshold for candidate gene research in our dataset. This threshold corresponded to *R*^2^ = 0.08 and was reached at a 99,885 bp distance on average. Therefore, a 100 kb genomic region was scanned in both directions from each significant SNP to look for candidate genes.

### 4.5. Genomic Selection

The SNPs in common between the GS training and validation sets, amounting to 5537, were employed to build GS models for grain yield, protein content, and protein yield based on ridge-regression BLUP [33]. Predictive ability values (computed as Pearson’s correlation between the observed phenotypic values and the breeding values predicted by GS) were estimated on the single RIL populations (within-population prediction), and on the pooled lines of the populations (across-population prediction) belonging to each GB (target and non-target GB).

## 5. Conclusions

In conclusion, our study (a) confirmed the polygenic control of pea grain yield and protein content, (b) indicated the possibility for joint improvement in both traits on the basis of the absence of inverse trait correlation, (c) encouraged the exploitation of GS for improvement of both traits in a target GB, while highlighting the appeal of GS for a non-target GB only for protein content improvement, and (d) revealed genomic regions of potential interest for the marker-assisted selection of both traits, albeit, for grain yield, partly influenced by the extent of cold stress in the specific cropping year.

## Figures and Tables

**Table 1:** Mean, phenotypic coefficient of variation (*CV*_1_ for 2018–2019 and *CV*_2_ for 2019–2020), and Pearson’s correlation (*r*) for grain yield (GY; t/ha), protein content (PC; %), and protein yield (PY; t/ha) of five Recombinant Inbred Line (RIL) populations, each represented by 23 lines.

		RIL Population					
Trait	Statistic	A × I	K × I	D × A	A × G	C × I	Mean
GY	Mean ^a^	6.20 a	6.18 a	5.46 b	5.36 b	5.12 b	5.66
GY	*CV*_1_	12.9	27.9	25.9	24.5	32.8	24.8
GY	*CV*_2_	16.4	17.5	18.6	26.9	23.2	20.5
GY	*r* ^b^	0.41 +	0.57 **	0.42 +	0.42 *	0.08 NS	0.38
PC	Mean ^a^	22.74 c	23.28 b	22.53 c	23.80 a	22.54 c	23.0
PC	*CV*_1_	4.2	6.0	6.1	7.0	6.5	6.0
PC	*CV*_2_	4.4	4.3	4.7	3.7	27.3	8.9
PC	*r* ^b^	0.49 *	0.65 ***	0.58 **	0.44 *	0.43 +	0.52
PY	Mean ^a^	1.42 a	1.46 a	1.24 bc	1.28 b	1.17 c	1.31
PY	*CV*_1_	13.6	30.9	26.9	24.9	34.1	26.1
PY	*CV*_2_	17.8	19.5	19.8	28.1	37.2	24.5
PY	*r* ^b^	0.50 *	0.60 **	0.49 *	0.52 *	0.21 NS	0.46

^a^ Different letters indicate significantly different means according to Duncan’s test (*p* < 0.05). ^b^ Difference from zero: + *p* < 0.10; * *p* < 0.05: ** *p* < 0.01; *** *p* < 0.001. NS: not significant.

**Table 2:** Genomic selection within- and across-population predictive ability values based on ridge-regression BLUP and 5537 SNPs. Validation was performed by using data from each of the two cropping years or their mean on a target genetic base (GB), including two Recombinant Inbred Line (RIL) populations, and a non-target GB, including three other RIL populations, each sharing one parent with the target GB.

		Predictive Ability				
		Within RILs			Across RILs	
Trait	Year	Target GB	Non-Target GB		Target GB	Non-Target GB
Grain yield	2018–2019	0.439	0.113		0.359	−0.087
Grain yield	2019–2020	0.458	0.011		0.525	−0.100
Grain yield	mean	0.505	0.079		0.480	−0.110
Protein content	2018–2019	0.534	0.372		0.560	0.295
Protein content	2019–2020	0.675	0.314		0.632	0.117
Protein content	mean	0.673	0.360		0.663	0.229
Protein yield	2018–2019	0.452	0.085		0.400	−0.155
Protein yield	2019–2020	0.490	−0.089		0.572	−0.269
Protein yield	mean	0.514	0.003		0.513	−0.256

Model training performed on 276 lines from three RIL populations; validation performed on 108 lines not included in the GS training set from the same or a different GB.

**Table 3:** Genomic selection within-population predictive ability values for each of five Recombinant Inbred Line (RIL) populations based on ridge-regression BLUP and 5537 SNPs. Validation was performed by using data from each of two cropping years or their mean, on a target genetic base (GB), including two RIL populations, and a non-target GB, including three other RIL populations, each sharing one parent with the target GB.

		Predictive Ability					
		Target GB			Non-Target GB		
Trait	Year	A × I	K × I		D × A	A × G	C × I
Grain yield	2018–2019	0.368	0.510		−0.063	0.147	0.256
Grain yield	2019–2020	0.303	0.613		−0.149	0.047	0.136
Grain yield	mean	0.407	0.603		−0.100	0.104	0.233
Protein content	2018–2019	0.575	0.492		0.202	0.721	0.195
Protein content	2019–2020	0.636	0.714		0.322	0.288	0.331
Protein content	mean	0.708	0.639		0.385	0.663	0.030
Protein yield	2018–2019	0.387	0.518		−0.094	0.111	0.237
Protein yield	2019–2020	0.319	0.662		−0.186	0.030	−0.111
Protein yield	mean	0.412	0.616		−0.167	0.074	0.101

Model training performed on 276 lines from three RIL populations; validation performed on 108 lines not included in the GS training set from the same or a different GB.

## Data Availability

The genotypic and phenotypic data used for this study are available in the Figshare repository with https://doi.org/10.6084/m9.figshare.28360028.

## Supplementary Materials

The following supporting information can be downloaded at: https://www.mdpi.com/article/10.3390/ijms26072991/s1.

## Abbreviations

The following abbreviations are used in this manuscript:

GEI	Genotype × environment interaction
QTL	Quantitative trait locus
GS	Genomic selection
GBS	Genotyping by sequencing
RIL	Recombinant Inbred Line
SNP	Single-Nucleotide Polymorphism
NIRS	Near-infrared spectroscopy
MAF	Minor allele frequency
