# Supplementary material for: Genomic Selection for Pea Grain Yield and Protein Content in Italian Environments for Target and Non-Target Genetic Bases
Source: Int J Mol Sci. 2025 Mar 25;26(7):2991. doi: 10.3390/ijms26072991 (PMC11988471; doi:10.3390/ijms26072991)

**Table S1.** Analysis of variance *F* test (fixed factors) or REML-likelihood ratio tests (random factors) results for grain yield, protein content, and protein yield per unit area of 115 pea inbred lines belonging to five Recombinant Inbred Line (RIL) populations evaluated for two cropping years in northern Italy.

| Source of variation | Grain yield | Protein content | Protein yield |
|---|---|---|---|
| Population | *** | *** | *** |
| Year | *** | *** | *** |
| Line | *** | *** | *** |
| Population x year | NS | NS | NS |
| Line x year | * | *** | * |

*, $p < 0.05$; ***, $p < 0.001$; NS, not significant ($p > 0.10$).

**Table S2.** Broad-sense heritability values for a genomic selection target and non-target genetic base (GB) in two cropping years for three pea traits.

| Dataset | Grain yield | Protein content | Protein yield |
|---|---|---|---|
| 2018-19, target GB | 0.54 | 0.85 | 0.62 |
| 2018-19, non-target GB | 0.67 | 0.91 | 0.71 |
| 2019-20, target GB | 0.67 | 0.74 | 0.75 |
| 2019-20, non-target GB | 0.77 | 0.84 | 0.79 |
| Mean | 0.66 | 0.84 | 0.72 |

**Table S3.** Significant markers detected by a GWAS based on 18,674 SNP and 276 pea lines belonging to three connected Recombinant Inbred Line populations for grain yield and protein content averaged across three test environments and grain yield in a mild-winter (Lodi 2013-14) and a cold-prone (Lodi 2014-15) environment, with the relative estimated effect.

| SNP | Trait | Effect |
|---|---|---|
| chr1LG6_167158777 | Grain yield; grain yield, Lodi 2014-15 | 0.29 |
| chr3LG5_110943532 | Grain yield | 0.36 |
| chr6LG2_112957117 | Grain yield | 0.30 |
| chr5LG3_555982018 | Grain yield; grain yield, Lodi 2014-15 | 0.33 |
| chr6LG2_72049750 | Grain yield | 0.26 |
| chr5LG3_548298174 | Protein content | 0.33 |
| scaffold00731_102978 | Protein content | 0.10 |
| chr1LG6_14126991 | Protein content | 0.30 |
| chr3LG5_122898115 | Protein content | 0.36 |
| chr5LG3_238119406 | Protein content | 0.36 |
| chr4LG4_7076997 | Protein content | 0.33 |
| chr5LG3_112688292 | Protein content | 0.37 |
| chr1LG6_55881393 | Protein content | 0.14 |
| chr2LG1_23689490 | Protein content | 0.34 |
| chr3LG5_13152476 | Protein content | 0.30 |
| chr6LG2_75499720 | Grain yield, Lodi 2013-14 | 0.33 |
| chr2LG1_293191007 | Grain yield, Lodi 2013-14 | 0.35 |
| chr6LG2_252910779 | Grain yield, Lodi 2013-14 | 0.28 |
| chr3LG5_302412301 | Grain yield, Lodi 2013-14 | 0.07 |
| chr3LG5_213572246 | Grain yield, Lodi 2014-15 | 0.36 |
| chr6LG2_78688716 | Grain yield, Lodi 2014-15 | 0.30 |
| chr5LG3_207358795 | Grain yield, Lodi 2014-15 | 0.28 |
| chr1LG6_195757998 | Grain yield, Lodi 2014-15 | 0.35 |
| chr2LG1_383235559 | Grain yield, Lodi 2014-15 | 0.36 |
| chr3LG5_109454037 | Grain yield, Lodi 2014-15 | 0.36 |

**Table S4.** List of genes potentially associated to significant SNP detected by a GWAS based on 18,674 SNP and 276 lines belonging to three connected Recombinant Inbred Line populations for grain yield and protein content averaged across three test environments. Candidate genes were identified by scanning a 100 kb region in both directions from each significant SNP and are reported with their annotated function (https://urgi.versailles.inra.fr/).

| Significant_SNP | Trait | Candidate_Gene | Function |
|---|---|---|---|
| chr1LG6_167158777 | Grain yield | Psat1g096760 | Phosphatidylethanolamine-binding protein |
| chr1LG6_167158777 | Grain yield | Psat1g096800 | TatD related DNase |
| chr1LG6_167158777 | Grain yield | Psat1g096840 | Major intrinsic protein |
| chr1LG6_167158777 | Grain yield | Psat1g096880 | GDA1/CD39 (nucleoside phosphatase) family |
| chr1LG6_167158777 | Grain yield | Psat1g096920 | Unknown gene |
| chr3LG5_110943532 | Grain yield | Psat3g051800 | Utp11 protein |
| chr3LG5_110943532 | Grain yield | Psat3g051840 | Zinc finger + C3HC4 RING-type |
| chr3LG5_110943532 | Grain yield | Psat3g051880 | RING-variant domain |
| chr3LG5_110943532 | Grain yield | Psat3g051920 | TPR repeat region circular profile |
| chr3LG5_110943532 | Grain yield | Psat3g051960 | PfkB family carbohydrate kinase |
| chr3LG5_110943532 | Grain yield | Psat3g052000 | Unknown gene |
| chr6LG2_112957117 | Grain yield | Psat6g081160 | Unknown gene |
| chr6LG2_112957117 | Grain yield | Psat6g081200 | Zinc-binding in reverse transcriptase |
| chr6LG2_112957117 | Grain yield | Psat6g081240 | Glutathione S-transferase + N-terminal domain |
| chr6LG2_112957117 | Grain yield | Psat6g081280 | Unknown gene |
| chr5LG3_555982018 | Grain yield | Psat5g289640 | Electron transfer flavoprotein-ubiquinone oxidoreductase + 4Fe-4S |
| chr5LG3_555982018 | Grain yield | Psat5g289680 | Regulation of cellular nucleobase + nucleoside + nucleotide and nucleic acid metabolic process |
| chr5LG3_555982018 | Grain yield | Psat5g289720 | Metallo-beta-lactamase superfamily |
| chr5LG3_555982018 | Grain yield | Psat5g289760 | BZIP transcription factor |
| chr6LG2_72049750 | Grain yield | Psat6g064240 | WD domain + G-beta repeat |
| chr6LG2_72049750 | Grain yield | Psat6g064280 | Protein of unknown function (DUF861) |
| chr6LG2_72049750 | Grain yield | Psat6g064320 | Beta-ketoacyl synthase + C-terminal domain |
| chr6LG2_72049750 | Grain yield | Psat6g064360 | Beta-ketoacyl synthase + N-terminal domain |
| chr6LG2_72049750 | Grain yield | Psat6g064400 | Zinc-binding dehydrogenase |
| chr6LG2_72049750 | Grain yield | Psat6g064440 | TPR repeat region circular profile |
| chr6LG2_72049750 | Grain yield | Psat6g064480 | Unknown gene |
| chr6LG2_72049750 | Grain yield | Psat6g064520 | ABC transporter |
| chr5LG3_548298174 | Protein content | Psat5g282440 | UAA transporter family |
| chr5LG3_548298174 | Protein content | Psat5g282480 | Homeobox' domain profile |
| chr5LG3_548298174 | Protein content | Psat5g282520 | Unknown gene |
| chr5LG3_548298174 | Protein content | Psat5g282600 | 3 +4-dihydroxy-2-butanone 4-phosphate synthase |
| chr1LG6_14126991 | Protein content | Psat1g010880 | Transferase activity + transferring phosphorus-containing groups |
| chr1LG6_14126991 | Protein content | Psat1g010920 | Cellular nucleobase + nucleoside + nucleotide and nucleic acid metabolic process |
| chr1LG6_14126991 | Protein content | Psat1g010960 | Serine/cysteine peptidase + trypsin-like |
| chr1LG6_14126991 | Protein content | Psat1g011000 | Serine/cysteine peptidase + trypsin-like |
| chr3LG5_122898115 | Protein content | Psat3g058360 | Unknown gene |
| chr3LG5_122898115 | Protein content | Psat3g058400 | AP2 domain |
| chr3LG5_122898115 | Protein content | Psat3g058440 | Ring finger domain |
| chr3LG5_122898115 | Protein content | Psat3g058480 | Autophagy protein Apg9 |
| chr3LG5_122898115 | Protein content | Psat3g058520 | Unknown gene |
| chr3LG5_122898115 | Protein content | Psat3g058560 | Myc-type + basic helix-loop-helix (bHLH) domain profile |
| chr3LG5_122898115 | Protein content | Psat3g058600 | HR-like lesion-inducing |
| chr5LG3_238119406 | Protein content | Psat5g132320 | LysM domain |
| chr5LG3_238119406 | Protein content | Psat5g132360 | Transcription factor Tfb4 |

| | | | |
|---|---|---|---|
| chr5LG3_238119406 | Protein content | Psat5g132400 | Transcription factor Tfb4 |
| chr5LG3_238119406 | Protein content | Psat5g132440 | Histone chaperone domain CHZ |
| chr4LG4_7076997 | Protein content | Psat4g006600 | Galactosyltransferase |
| chr4LG4_7076997 | Protein content | Psat4g006640 | Unknown gene |
| chr4LG4_7076997 | Protein content | Psat4g006680 | Methyltransferase TYW3 |
| chr5LG3_112688292 | Protein content | Psat5g062600 | NYN domain |
| chr5LG3_112688292 | Protein content | Psat5g062640 | EamA-like transporter family |
| chr5LG3_112688292 | Protein content | Psat5g062680 | Unknown gene |
| chr5LG3_112688292 | Protein content | Psat5g062720 | Triose-phosphate Transporter family |
| chr5LG3_112688292 | Protein content | Psat5g062760 | ABC transporter |
| chr5LG3_112688292 | Protein content | Psat5g062800 | Cyanobacterial and plant NDH-1 subunit O |
| chr1LG6_55881393 | Protein content | NA | NA |
| chr2LG1_23689490 | Protein content | Psat2g022120 | FAM91 N-terminus |
| chr2LG1_23689490 | Protein content | Psat2g022160 | Leucine rich repeat N-terminal domain |
| chr2LG1_23689490 | Protein content | Psat2g022240 | Clathrin adaptor complex small chain |
| chr2LG1_23689490 | Protein content | Psat2g022280 | Intracellular membrane-bounded organelle |
| chr2LG1_23689490 | Protein content | Psat2g022320 | Ethylene insensitive 3 |
| chr3LG5_13152476 | Protein content | Psat3g004240 | BTB/POZ domain |
| chr3LG5_13152476 | Protein content | Psat3g004280 | Unknown gene |
| chr3LG5_13152476 | Protein content | Psat3g004320 | BTB/POZ domain |
| chr3LG5_13152476 | Protein content | Psat3g004360 | BTB And C-terminal Kelch |
| chr3LG5_13152476 | Protein content | Psat3g004400 | BTB/POZ domain |
| chr3LG5_13152476 | Protein content | Psat3g004440 | BTB/POZ domain |

**Table S5.** Sowing date and value of climatic variables during the cropping years of genomic selection (GS) training and validation experiments.

| Item | Sowing date | Rainfall (mm) | Abs. min. daily T (°C) | No. frost days |
|---|---|---|---|---|
| **GS training experiments** | | | | |
| Lodi 2013-14 | 07/11/2013 | 465 | –5.7 | 35 |
| Perugia 2013-14 | 25/11/2013 | 459 | –3.6 | 9 |
| Lodi 2014-15 | 22/10/2014 | 345 | –11.6 | 34 |
| **GS validation experiments** | | | | |
| Lodi 2018-19 | 25/10/2018 | 308 | –12.0 | 54 |
| Lodi 2019-20 | 10/12/2019 | 192 | –10.9 | 34 |

**Figure S1.** Manhattan plots showing the association scores of 18,674 SNP along the 7 pea chromosomes (chromosome 8 represents scaffolds) with grain yield in two test environments. The GWAS was based on Blink model and 276 lines belonging to three connected Recombinant Inbred Line populations. The continuous and dashed lines represent Bonferroni and False Discovery Rate thresholds, respectively, at $p < 0.01$.

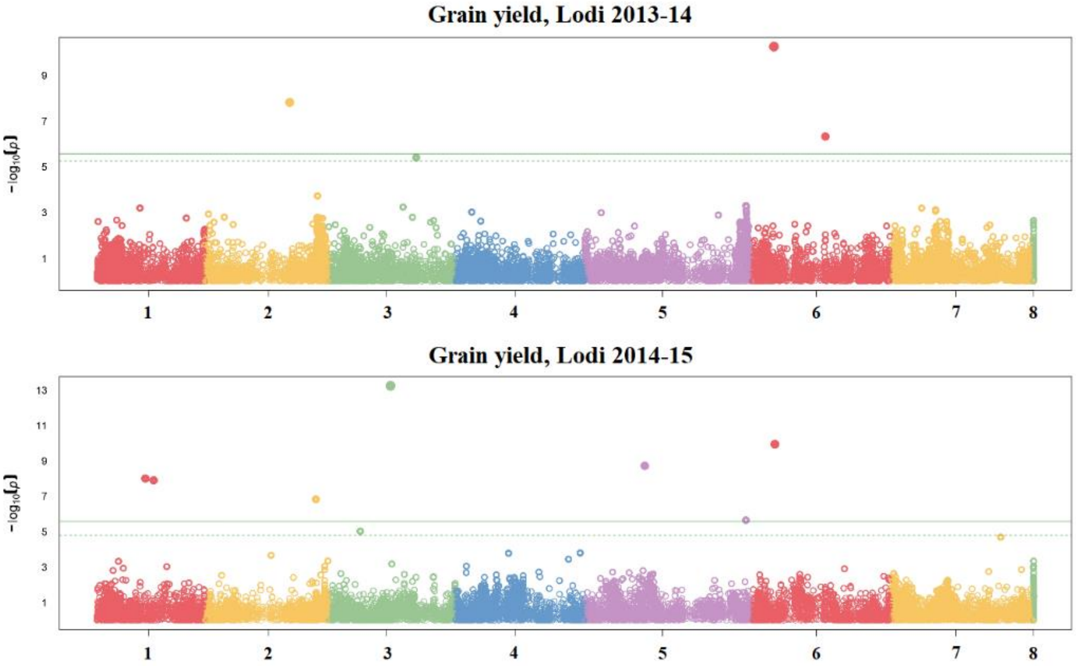


**Figure S2.** Quantile-Quantile plots of expected vs. observed association scores of 18,674 SNP for two pea traits and 276 lines belonging to three connected Recombinant Inbred Line populations. The red line represents equality between the expected and observed quantiles and the grey area the associated 95% confidence interval. The two upper plots refer to mean trait data across three environments, while the lower two to data from two single environments.

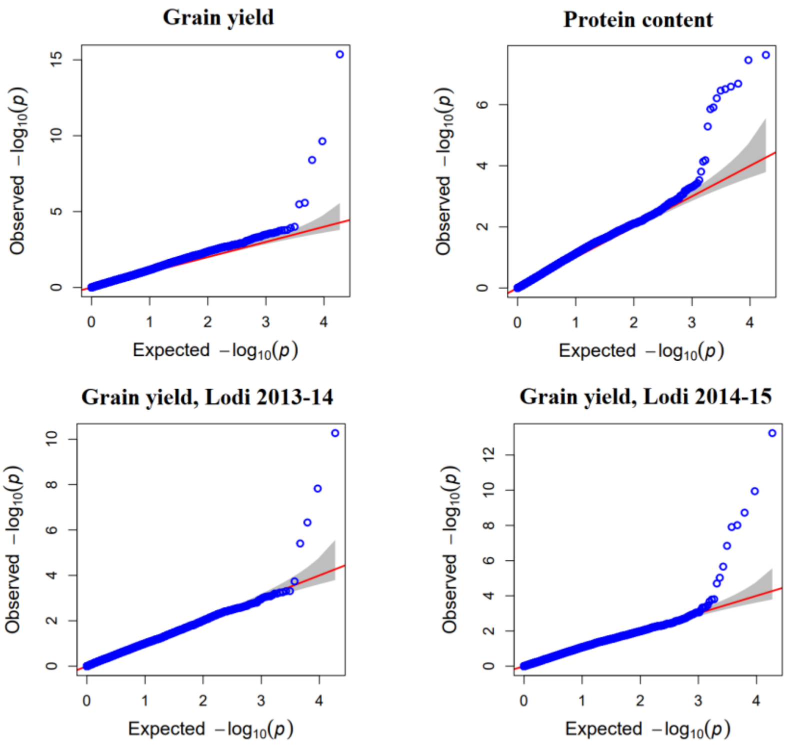

Supplement: Supplementary file 1 [file ijms-26-02991-s001.zip › ijms-3491782-supplementary.pdf]
